# Remote Learning and Its Impact on Newly Matriculated Medical Students

**DOI:** 10.7759/cureus.17223

**Published:** 2021-08-16

**Authors:** Nicholas B Conway, Helen G Tempest, Jenny Fortun

**Affiliations:** 1 Office of Medical Education, Florida International University, Herbert Wertheim College of Medicine, Miami, USA; 2 Department of Human and Molecular Genetics & Biomolecular Sciences Institute, Florida International University, Herbert Wertheim College of Medicine, Miami, USA; 3 Department of Cellular Biology and Pharmacology, Florida International University, Herbert Wertheim College of Medicine, Miami, USA

**Keywords:** online medical education, covid-19, assessment in health professions education, medical education assessment, medical education research

## Abstract

Objective

The coronavirus disease 2019 (COVID-19) pandemic has led to massive disruptions in medical education. In the fall of 2020, newly matriculated medical students around the country started medical school in a remote learning setting. The purpose of this study is to assess the impact of remote learning during the COVID-19 pandemic on academic performance and student satisfaction among first-year medical students.

Methods

The newest cohort of first-year medical students (class of 2024; n = 128) who completed their first basic science course, "Genes, Molecules & Cells (GMC)," using an adapted remote format was compared to the prior year's cohort (class of 2023; n = 122) of first-year medical students who were taught using traditional approaches. The items that were compared were numerical performance on exams and quizzes, study strategies, and course evaluation in GMC. Data were analyzed with a two-sided *t*-test and Pearson correlation coefficient. Students' perception of remote learning was also reported and results were obtained using a five-point Likert scale through anonymous surveys via E-value.

Results

No statistical difference was observed in students' performance on the midterm and final examinations between the two cohorts in both multiple-choice and written examinations. Mean multiple-choice question (MCQ) midterm students' performance in remote learning compared to traditional learning cohort was 75.9%, standard deviation (SD) 6.1 to 75.89%, SD 6.49, respectively. Mean MCQ final students' performance was 84%, SD 6.37 (class of 2024) to 85%, SD 8.78 (class of 2023). Students’ satisfaction with their learning experience was similar among the two groups (class of 2024: mean = 4.61, SD 0.66; class of 2023: mean = 4.57, SD 0.68). Most students (70%) in the remote learning cohort had a positive opinion of remote learning. Of the students, 17% reported feeling disconnected, isolated, or not actively involved.

Conclusions

The results of this study demonstrate that not only is remote learning effective but that the students were also resilient in their adaptation to a new learning format. Our experience highlights the importance of including wellness solutions to mitigate the feeling of isolation and disconnection during remote learning.

## Introduction

The novel coronavirus disease 2019 (COVID-19), since first being reported in the United States in January of 2020, has had a tremendous impact on all aspects of society [1]. One field that has been radically impacted is higher education, specifically medical education [2]. In March of 2020, after the dramatic increase in cases and, at the time, limited information on the pathogenesis of COVID-19, the Florida International University (FIU) Herbert Wertheim College of Medicine (HWCOM) rapidly transitioned to remote learning for students based on the Association of American Medical Colleges (AAMC) recommended guidelines [3]. This transition has not been short-lived, with many students nationally starting medical school remotely. The potential impact of this transition on academic performance is yet to be described.

The use of remote/e-learning in medical education has seen a dramatic increase in utilization in recent years [4]. With the COVID-19 pandemic forcing education out of the traditional classroom setting and into more virtual settings, it is likely this trend will continue to grow exponentially. Commonly, remote learning is used in combination with traditional face-to-face interactions to enhance the learning experience for medical students; however, its use is highly variable from program to program [5]. Internationally, programs in developing countries that have limited access to faculty have utilized remote teaching methods more extensively to improve access to education [6].

There is currently limited literature on the effects of using remote learning exclusively on student performance in assessments. Frehywot et al. in 2013 conducted an analysis of existing articles from international medical programs showing pure e-learning environments compared to traditional educational environments and showed favorable results as a method to teach medical knowledge [6]. An important distinction to make is that these courses were designed for online learning from the outset, as opposed to courses that are traditionally taught face-to-face and had to rapidly transition to a remote platform. Courses designed for a meaningful online experience are carefully crafted and should be evaluated differently [7]. A small randomized control trial (RCT) in 2016 comparing face-to-face and remote-online case-based discussions demonstrated comparable performance on multiple-choice question (MCQ) exams between the two groups [8]. A meta-analysis examining remote learning in nursing school demonstrated e-learning methods outperformed that of the traditional lecture format. This meta-analysis is limited in the fact that they did not perform meta-regression to determine plausible explanations for their findings. However, many of these studies used a blended model rather than remote learning alone [9].

While remote learning has existed for many years in medical education, it has never been implemented on such a grand scale. It presents new and unique opportunities as well as challenges for both educators and students alike, as many programs rapidly had to transition to fully remote learning. Currently, programs both nationally and internationally are stepping up to meet these challenges [3]. Medical education has continuously been at the forefront of adopting new and innovative educational pedagogies [4]. Due to this unprecedented moment, there are rightfully some concerns about the potential effects of this transition on students [3]. One of these concerns is how students will perform when compared to previous cohorts. The disadvantages of remote learning have been well documented in educational theory, and these include: certain student populations (men, Black students, younger individuals) had difficultly in transitioning to remote learning [10] and had statistically significant low grand-point ages compared to peers, poor student engagement [11], less opportunity for student engagement, and technical/internet bandwidth limitation [12].

Here at HWCOM, we welcomed our newest cohort of first-year medical students virtually. This group completed their first basic science course entirely using remote learning. We sought to explore how this cohort of students compared to a prior cohort who completed the course face-to-face, in terms of their academic performance on critical thinking exams (CTEs) containing open-ended questions (OEQ) and MCQ exams and quizzes, both faculty-generated and questions selected from the National Board of Medical Examiners (NBME), as well as student satisfaction.

## Materials and methods

Groups

Genes, Molecules, and Cells (GMC) was the first basic science course at the HWCOM. The GMC course was designed to introduce the fundamental concepts of cell and molecular biology, biochemistry, and medical genetics as they relate to normal and disease processes. This study compared two cohorts. Group A (academic year 2019, 128 students) received the traditional GMC course with in-person lectures and active learning activities. In-person attendance was not required for all lectures but was required for active learning sessions. The GMC course was adjusted during the academic year 2020 (group B, 122 students) and completely transitioned to remote learning due to the COVID-19 pandemic. For group B, all course activities were remote and conducted live via Zoom (Zoom Video Communications, Inc., San Jose, California). The course length (eight weeks), content, learning objectives, and pedagogies were the same for both cohorts. This study was exempted from IRB from the local institutional review board (IRB-21-0034). Class demographics were similar for both cohorts (Table 1). 

**Table 1:** Demographics for group A (class of 2023) and group B (class of 2024) MCAT - Medical College Admission Test.

	Group A	Group B
Females	57%	53%
Male	43%	47%
White (Non-Hispanic)	35%	27%
Hispanic/Latino	34%	27%
Asian	17%	26%
Black (Non-Hispanic)	7%	11%
Average MCAT	508	509

Evaluations

Both groups were tested using a combination of MCQ and CTEs containing OEQ. Two CTEs written by course faculty were administered at the midpoint (midterm) and end (final) of the course. The goal of these written exams is to evaluate the ability of students to explain complex problems (e.g., correlate signs and symptoms with a biochemical defect), to apply knowledge (e.g., propose and interpret diagnostic tests), and integrate among multiple material and disciplines of the course (e.g., explain the mechanism of disease or possible etiologies of presentation) [13,14]. The midterm MCQ exam was developed by course faculty and reviewed by three faculty outside the course and a copy editor. The final cumulative MCQ exam was selected from the NBME databank. All questions are mapped to the educational program and course learning objectives. A weekly checkpoint quiz (CPQ) was administered online each Monday (except midterm week). The CPQ consisted of 10-15 application and recognition questions related to the material learned in the preceding week. The grade distribution/grading scheme was similar with small differences between the two cohorts (Table 2). Quiz and exam questions were the same with the following two exceptions: three questions in the midterm were different questions and there was an additional CPQ for group B. Midterm and CTE exams were administered using ExamSoft for group A and CanvasMed with HonorLock for group B, under standard closed book exam conditions (no access to study resources or online search engines, blank scratch paper, and proctors present). Both student populations received training in either ExamSoft or CanvasMed with Honorlock. Group A students received feedback immediately after the midterm and CTE exams, but group B did not due to the different functionalities of the software. Student grades in exams were provided by the Office of Assessment. Student grades in quizzes were collected directly from CanvasMed.

**Table 2:** Grade distribution/grading scheme for the traditional learning methods cohort (A) and remote learning cohort (B) IRAT: Individual Readiness Assurance Test; CTE: critical thinking exam; MCQ: multiple-choice question; CPQ: checkpoint quiz; FC: flipped classroom; CBL: case-based learning.

	Mock CTE	Midterm CTE	Midterm MCQ	Final CTE	Final MCQ	FC	IRAT	CBL	CPQ
A	1	7	30	10	50	1	0	0	1
B	1	8	25	10	40	5	5	1	5

Surveys

All surveys were anonymous. Surveys were administered at the end of the course for both groups. The surveys contained two types of questions: questions using a five-point Likert scale (5, strongly agree; 1, strongly disagree) and OEQs (Appendix A). For all questions, students had the opportunity to provide additional written feedback. Data were collected by the Office of Assessment (independent from course faculty). Each survey was standardized and used in previous iterations of the course. Additional questions were added to assess remote learning impact (Appendix B). Written comments about their experience with remote learning and study habits were categorized and tabulated by blinded reviewers.

Statistical analysis

Differences between scores of the two groups were evaluated using a Student's two-sided t-test. The correlation between two sets of data was evaluated by the Pearson correlation coefficient. Statistical significance was considered α = 0.05. Analysis was conducted using IBM’s Statistical Package for Social Sciences (SPSS) V26 Statistics (IBM SPSS Statistics, Armonk, New York) software version.

## Results

All lectures and activities (except the case-based learning [CBL]) were recorded and were available to students within 24 h for both cohorts. The total duration of students listening to recordings in the course was 2,839 h and 2,506 h for groups A and B, respectively. The estimated usage per student throughout the course was about the same (22 times in group A and 21 times in group B).

We did not observe any statistical differences between the two cohorts in any of the CTE or MCQ exams. Mean performance on midterm MCQ/CTE was 75.9% (standard deviation (SD) 6.1%)/64.7% (SD 4.25) and 75.98% (SD 6.49%)/62.8% (SD 4.26%) for groups A and B, respectively. Final MCQ/CTE performance for groups A and B were 84% (SD 6.37%)/79.7% (SD 3.25%) and 85% (SD 8.78%)/81.7% (SD 3.26%), respectively. Final average course grades were 87.2% (SD 6.4%; group A) and 86.6% (SD 8.09%; group B). We did not see any trend of increased or decreased performance on identical CTE questions. Differences in questions ranged between 0.02 and 0.67 points, with an average difference of 0.28 points among cohorts, none of which was significant (Table 3).

**Table 3:** Assessments and students' performance for the traditional learning methods cohort (A) and remote learning cohort (B) MCQ: multiple-choice question; CTE: critical thinking exam; OEQ: open-ended question; NBME: National Board of Medical Examiners; SD: standard deviation; KR-20: Kuder-Richardson 20.

Assessment category	Statistical parameter	A	B
Midterm MCQ (in-house)	Mean correct	75.90	75.89
	N	61	60
	SD	6.10	6.49
	KR-20	0.77	0.81
Midterm CTE N = 6 OEQ	Mean correct	64.7	62.8
	SD	4.25	SD 4.26
Final MCQ (NBME) N = 120 MCQ questions Item difficulty 0.72	Mean correct	84	85
	SD	6.37	8.78
	Discrimination difficulty	0.25	0.32
Final CTE N = 6 open-ended questions	Mean correct	79.7	81.70
	SD	3.25	3.26
Final course grade	Mean correct SD	87.2 6.4	86.6 8.09

In addition to CTE and MCQ exams, quizzes contributed to the grades for both cohorts, i.e., CPQ and flipped classroom (FC) quizzes. To facilitate comparison, all quizzes were converted to 100%. The mean grade of FC quizzes for group B was slightly higher than group A (87% vs. 84%, *p *< 0.05; Table 3). A similar trend was seen for the mean CPQ (88% for group B vs. 85% for group A; Table 3).

As part of our standard quality improvement procedures, students complete an anonymous course evaluation at the end of the course. The response rate for group A was 89.8% (115/128) and 91.8% (112/122) for group B. The level of satisfaction with the course based on the student survey was similar between cohorts (Table 4). Most students were very or extremely satisfied with all pedagogies, with a stronger preference for lectures (39%) and FC (29%) sessions, followed by CBL (12%) and formative quizzes (10%).

**Table 4:** Course evaluation for the class of traditional learning methods cohort (group A) and remote learning (group B)

	Group A (n = 115)		Group B (n = 112)	
	Mean	Std	Mean	Std
The lecture content matched the learning objectives	4.63	0.6	4.65	0.58
The assignments or activities were relevant and supported the course content	4.57	0.66	4.56	0.67
The course was well organized	4.52	0.76	4.49	0.81
The course director communicated effectively with students and faculty	4.67	0.63	4.55	0.70
This course fostered my learning	4.64	0.62	4.61	0.66
Average	4.61	0.66	4.57	0.68

Satisfaction with remote learning (group B only) was evaluated with specific questions using a Likert scale and via a qualitative tabulation of comments to the prompt “Comment on your remote learning experiences in this course.” Most students were very or extremely satisfied with the remote experience and all the learning activities in the course (Table 5). In agreement with their preference, students found the delivery of lectures via Zoom was the most effective learning format, followed by Individual Readiness Assurance Test (IRAT)/group discussion, FC, and CBLs.

**Table 5:** Students' satisfaction with remote learning (group B) IRAT: Individual Readiness Assurance Test; SD: standard deviation.

Group B		Mean	SD
Lectures delivered by Zoom were effective	110	4.45	0.75
IRAT/group discussions delivered by Zoom were effective	112	4.38	0.8
Flipped classrooms delivered by Zoom were effective	111	4.29	0.87
Case-based learning delivered by zoom was effective	112	4.18	1.03
I had access to all the resources I needed to study effectively in the remote environment	112	4.27	0.97
How satisfied were you overall with the course being run by remote learning	112	4.25	0.99

## Discussion

The COVID-19 pandemic has dramatically affected medical education as numerous programs across the country have welcomed students in new and unfamiliar ways [2]. Educators have been challenged to rapidly innovate to deliver the same content to students in unfamiliar ways [3]. In this paper, we demonstrated that there were no differences in numerical performance on identical questions between remote and traditional learning. First-year medical students start their training at HWCOM with multiple courses but this study focused on GMC, their first basic science course. GMC is a course that challenges students to apply knowledge from biochemistry, genetics, and molecular and cell biology to reason through mechanisms of disease and interpret symptoms, signs, and laboratory data. To accomplish this, a variety of active learning pedagogies are incorporated where the students are asked to reflect on a task or a clinical problem and discuss their ideas with their peers. Accordingly, students are tested using exams that evaluate these skills [13,14]. We were not surprised by the lack of statistical differences in performance given that we maintained the structure and integrity of the course, including learning objectives, pedagogies, resources, and types of assessments. While the mean grades on FC quizzes and CPQs were slightly higher in group B when compared to group A, these small increases might have been influenced by a larger contribution of FC quizzes and CPQ to the final grade distribution in group B. An important limitation of this study is that it only includes a small sample size occurring at one time point (one class) at only one medical school. Thus, the results might not be applicable to all courses in medical education or at our school, more specifically those related to direct patient contact. While a randomized control trial would be the gold standard, it was not feasible nor equitable in the current pandemic. Although given the cross-sectional nature of the study, we cannot predict the trend observed in this study will continue through future foundational courses; however, we believe it will be, if students are provided with adequate resources.

**Figure 1:**
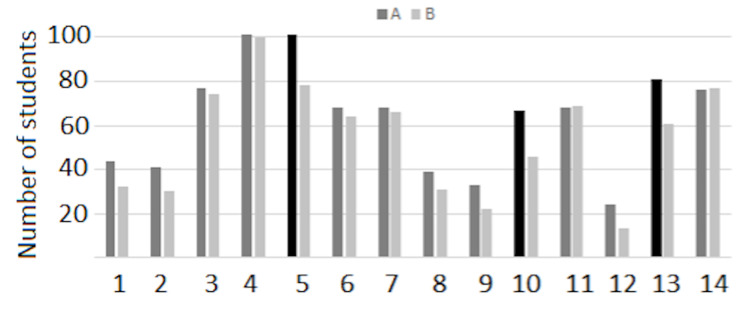
Results of students' self-reported study strategies utilized to prepare for the CTE exam for group A and group B The number of students reporting using different studying strategies was counted for groups A (dark grey) and B (light grey). Strategies tabulated were: constructing diagrams or concept maps about diagnosis covered in class (1); or connecting signs/symptoms with molecular and cellular bases of diseases (2); focusing on "why" questions more than "what" questions (3); connecting disease presentation to signs/symptoms and diagnostic markers (4); understanding more than memorizing (5); identifying diagnosis covered in class and memorizing all the information (6); looking at the big picture and concentrating later in details (7); identifying or making cards of different diagnosis with the main points for each one (8); or making cards starting with signs and adding the different diagnosis associated with each sign (9); organizing knowledge by disease presentation, as opposed to isolated concepts/disciplines (10); reading about diagnosis covered in class (11); studying content in class without concentrating in specific diagnoses (12); studying in groups and verbalizing explanations (13); and watching videos about diagnosis covered in class (14). More students in group A employed strategies 5, 10, and 13 (black bars), compared to group B. CTE: critical thinking exam.

We have employed a combination of MCQ exams and CTEs for several years. We have noticed a small to moderate correlation between CTE and MCQ exams, suggesting that the two types of exams may be testing different cognitive skills. Interestingly, the correlation coefficient between MCQ exams and CTEs was higher for group B, compared to group A (Table 6). Students reported using different strategies when studying for CTEs, as compared to MCQ exams. When studying for CTE exams, both cohorts self-reported focusing on understanding and integrating concepts in the context of disease presentation, as opposed to memorizing (Figure 1). The most pronounced differences between cohorts was a reduction in the number of students in group B (compared to A) focusing on understanding more than memorizing (78 vs. 101 students; Figure 1, category 6), organizing knowledge by disease presentation, as opposed to isolated concepts/disciplines (10; 46 vs. 67 students), studying in groups and verbalizing explanations (14; 61 vs. 81 students).

**Table 6:** Correlation between critical thinking exams (CTEs) and multiple-choice questions (MCQs) among groups A and B *** signifies *p* < 0.001.

Assessment category	Statistical parameter	A	B
Correlation midterm MCQ and CTE	Pearson	0.419***	0.602***
	R-square	0.167***	0.362***
Correlation final MCQ and CTE	Pearson	0.416***	0.614***
	R-square	0.173***	0.377***

While students in both cohorts focused on connecting disease presentation to signs/symptoms and diagnostic markers, students in the remote learning environment appeared to have focused more on memorizing and less on understanding and organizing knowledge by disease presentation. Unsurprisingly, group study was less employed by students in the remote learning environment compared to students in the traditional learning environment. This is concerning as the utilization of study groups among students in higher education has been associated with positive learning outcomes, as well as social outcomes [15]. Some students reported feeling disconnected or isolated in the remote learning environment (Table 7). The latter may explain the former. When understanding a topic, it is essential to discuss and have the opportunity to explain it to colleagues [16]. It is possible that the absence of discussion with, and input from, classmates resulted in students focusing more on lower-level learning objectives. This study does not address the potential differences in study habits or strategies between cohorts based on their prior experiences. Nonetheless, we do not believe demographics played a significant role given the similarities between cohorts (with the only noteworthy difference being an increase in Asians and African Americans in group B). One potential confounder that should be mentioned is the difference in the testing environment, while both were under standard closed book conditions, different testing environments do introduce more variability in the testing situation. We do believe this is negligible as it would have likely shown students in group B outperforming group A.

**Table 7:** Students' experience of remote learning: categorization of students' open-ended responses (group B)

Categories	Percent of responses	Categories	Percent of responses
Overall positive impression	70	Overall negative impression	13
Enjoyable course	38	Feelings of disconnection or isolation	17
Unhindered learning	31	Technical issues	13
Faculty appreciation	21	Difficulty adjusting	13
Flexible schedule	15	Hindered learning	7
Organized course	13		
Engaged sessions	10		

Most of the students in this study were satisfied with their remote learning experience. Remote learning may offer some advantages, such as increased flexibility. For example, the option of Zoom lectures may reduce their commute, increase the amount of participation by allowing them to use the chat option to ask or answer questions (perhaps more willingly than in person); in addition, some students may choose to watch the lecture at their own pace at a time they feel they are most productive. We did not quantify the attendance difference between the two cohorts. Typically as this is the first basic science course, the attendance is very high in person. On the other hand, some students felt disconnected or isolated and had difficulty adjusting to remote learning (Table 7). The wellness of students should be of utmost importance and ought to be a priority among all medical schools, especially given the current times. In 2012, Dyrbye et al. reported 55.9% of US medical students in their study experienced burnout, and a recent meta-analysis demonstrated the burden of burnout on both US and international medical students [17,18]. Our school offers robust counseling and wellness services including individual counseling and aromatherapy sessions, as well as numerous fit and well events such as yoga and stress management. There are also opportunities for mentorship from upper classmates through our leadership development program MedLEAD. We have an entire department, Student Academic Support Services (SASS), that offers a robust peer-tutoring service (large-group, small group, and one-on-one) that was completely remote during GMC. All of the above-listed resources were available to both cohorts (groups A and B). However, one new resource our SASS department created for group B was virtual study rooms to stimulate group studying while maintaining social distance and Centers for Disease Control and Prevention (CDC) guidelines. These study rooms and many of the wellness workshops are completely peer-led to encourage student turnout and involvement. It is important to create the conditions where these services can be not only maintained but expanded to accommodate the new challenges. While many students might be apprehensive, we believe that if these services are encouraged and normalized by faculty/staff as well as their peers, it will increase student utilization of these vital resources. It is essential to determine what is the long-term impact of feelings of isolation and reduced interactions with peers in student burnout and academic performance.

Medicine is an evidence-based art, medical education should be as well. It is vital that students be able to learn and master the requisite material to become future physicians. Here we provide data that demonstrated medical students in a remote setting are able to synthesize the necessary material and perform well on both faculty-generated and NBME examinations. We can infer from these data that even in the utmost uncertain times, both medical educators and students have been able to adapt ensuring that the students are still learning the material they will need to be the next generation of healthcare providers. Future directions include expanding on the data we reported here to see if this trend continues across the entire first period of medical education at HWCOM.

## Conclusions

Even in the face of the unprecedented challenges brought by the COVID-19 pandemic, students are able to learn the material and perform admirably on examinations while having an overall positive opinion of remote learning. However, remote learning can have negative implications including students feeling more isolated and disconnected.

## Appendices

Appendix A: Course Evaluation Questions/Statements for Both Group A and Group B

1. The lecture content matched the learning objectives.

2. The assignments or activities were relevant and supported the course content.

3. The course was well organized.

4. The course director communicated effectively with students and faculty.

5. This course fostered my learning.

6. Additional comments:

Appendix B: Additional Course Evaluation Questions/Statements for Remote Learning Students (Group B)

1. How satisfied were you overall with the course being run by remote learning?

2. Lectures delivered by Zoom were effective.

3. Application exercises delivered by Zoom were effective.

4. Flipped classrooms delivered by Zoom were effective.

5. Case-based learning sessions delivered by zoom were effective.

6. I had access to all the resources I needed to study effectively in the remote environment.

7. Which of these online instructional methods worked best for you? Lectures, flipped classroom, case-based learning, application exercises, checkpoint quizzes, formative quizzes, discussion boards, office hours, or question and answer sessions.

8. Comment on your remote learning experiences in this course:
